# Genome-Wide Identification and Analysis of the MADS-Box Gene Family in *Tectona grandis* (Teak), a Member of the Lamiaceae Family

**DOI:** 10.3390/genes17020124

**Published:** 2026-01-25

**Authors:** Tareq Alhindi, Khaldoun J. Al-Hadid, Ayed M. Al-Abdallat

**Affiliations:** 1 Department of Biological Sciences, School of Science, The University of Jordan, Amman 11942, Jordan; kalhadid@ju.edu.jo; 2 Department of Horticulture and Crop Science, School of Agriculture, The University of Jordan, Amman 11942, Jordan; a.alabdallat@ju.edu.jo

**Keywords:** teak, genome-wide analysis, Lamiaceae, MADS-box gene family, transcription factors, protein–protein interaction

## Abstract

Background: In plants, members of the MADS-box gene family encode transcription factors that regulate a wide range of developmental processes, including cell differentiation, organ identity, floral induction, and responses to environmental stimuli. Moreover, MADS-box genes play central roles in the well-known ABCDE model of floral development. Teak (*Tectona grandis*), a woody species belonging to the Lamiaceae family, is recognized for its medicinal and agricultural significance. The recent availability of a chromosome-level genome assembly for *T. grandis* has enabled the genome-wide identification of 87 MADS-box genes, which are distributed across 18 pseudo-chromosomes. Methods: The amino acid sequences of these genes were compared with orthologous proteins from *Arabidopsis thaliana*, *Sesamum indicum*, and *Amborella trichopoda* to infer the phylogenetic relationships. The structures of key floral quartets in the MADS-box proteins were predicted, and the stability of these predicted tetramers were analyzed via molecular dynamics simulations. Results: The phylogenetic analysis classified the genes into 33 Type I and 54 Type II MADS-box members, forming four major clades (MIKC^C^, MIKC*, Mα, and Mγ), while the Mβ-type clade was absent. A conserved motif analysis revealed that the Type II genes exhibited greater motif diversity than the Type I, suggesting that *T. grandis* Type II MADS-box genes possess more complex structures and potentially broader functions. The transcriptomic data from different tissues showed that the MIKC-type genes were particularly active during flower development. Although stable over the simulation time, the *T. grandis* AP3 ortholog had shorter I and K domains and had an odd mode of protein–protein interaction. Conclusion: Overall, the presented genome-wide analysis provides a comprehensive base for understanding the evolutionary diversification of the MADS-box gene family in *T. grandis* and identifies candidate genes for future structural and functional characterization.

## 1. Introduction

The mint family (Lamiaceae) contains numerous aromatic species, such as lavender, basil, rosemary, sage, and mint. Members of this family are typically characterized by having opposite leaves, bilaterally symmetric flowers, and square stems. Plants of this family are widely valued for their culinary, medicinal, and ornamental uses due to their essential oils and fragrant properties [1,2]. *T. grandis* (teak) belongs to this family, is native to a few countries in South and Southeast Asia, and is a prominent timber species that can reach a height of 25–30 m [3]. Teak is highly valued for its exceptional durability, dimensional stability, and resistance to decay [4].

Phytochemical studies have shown that teak tissues contain various phytochemicals, including naphthoquinones, anthraquinones, and isoprenoid quinones [5], which contribute to its medicinal properties, and are associated with anti-inflammatory, antioxidant, wound-healing, and antimicrobial activities [6,7]. Moreover, it has analgesic and hypoglycemic effects [5,8]. Teak extracts have traditionally been used to treat various ailments, including bronchitis, dysentery, and diabetes [9]. Recent studies have explored its potential in modern medicine, including its antidiuretic and hair growth-promoting activities [8,9]. Teak presents a promising source for developing new therapeutic agents. Therefore, various genetic improvement programs have been implemented to enhance both the productivity and quality of teak [10].

Genome-wide analyses of teak have revealed significant insights into its genetic structure and wood formation processes. A high-quality chromosomal-scale genome assembly has been developed, comprising 18 pseudo-chromosomes and 31,168 genes encoding 46,826 gene models [11,12]. The genome contains numerous simple sequence repeats (SSRs), which have an important role in genetic conservation and breeding programs [13]. In addition, 130 NAC transcription factors have been identified and analyzed for their potential role in wood formation and secondary cell wall synthesis [14]. These genomic resources are expected to enable the discovery of novel genes associated with desirable traits and to support sustainable teak production and conservation efforts. Genetic studies of teak have revealed moderate genetic diversity within populations and significant differentiation among populations [15,16]. High-throughput genotyping and microsatellite markers have been used to assess its genetic structure and diversity [17,18]. Furthermore, genome analyses have also uncovered the genes associated with natural products biosynthesis (e.g., terpene synthases [12]) and drought-stress responses [19], as well as correlations between photosynthetic traits and growth performance [20]. These genetic studies have important applications in teak improvement, including the selection of superior clones for plantation forestry and the development of conservation strategies [16,21].

MADS-box transcription factors constitute a conserved gene family that are widely distributed among eukaryotes lineages, including plants and animals [22]. They encode transcription factors crucial for developmental transitions and floral organogenesis and flowering behavior [22,23,24]. Phylogenetic analyses, gene structure investigations, and expression profiling have revealed the diverse functions of MADS-box genes across different species. Each MADS-box protein contains a highly conserved 56-amino-acid DNA-binding domain that mediates dimerization and binding to target promoters [22]. In plants, these genes are organized into distinct functional groups, such as AGAMOUS and APETALA, which diversified rapidly before the emergence of angiosperms [24]. Expression analyses have revealed tissue-specific patterns and involvement in multiple developmental processes and stress responses, making them potential targets for improving plant characteristics [25,26,27]. Comparative genomics has shown that MADS-box gene numbers vary widely across species, from 24 in eucalyptus [25] to 300 in bread wheat [28,29], with 211 in maize [27], 144 in radish [23], 62 in melon [30], 102 in coffee [31], 153 in potato [32], 90 in grapevine [33], and 114 in flax [34]. These studies have highlighted both conserved subfamilies and species-specific expansions to better understand their evolution and role in plant growth and development. Some subfamilies remain conserved across multiple species, while others have expanded uniquely in certain lineages [23,26,27,30].

MADS-box genes are typically divided into two major groups, Type I and Type II, that can be further divided into subgroups [23,27]. The type I MADS-box genes have been characterized in various plant species, including barley and *Brassica oleracea* [35,36]. The members of this group have been further categorized into three subgroups: Mα, Mβ, and Mγ [37]. Members of the Type I group usually play essential roles in female gametophyte development, as well as in embryo and seed formation [38,39]. In barley, the Type I MADS-box genes are predominantly expressed in the antipodal cells, the central cells, and chalazal endosperm [35]. Moreover, Type I MADS-box genes are subject to epigenetic regulation, including DNA methylation and histone modification [35]. The Type I genes are numerous in angiosperms but are less common in gymnosperms, suggesting an expansion of this gene family during angiosperm evolution [40].

In contrast, Type II MADS-box members (synonymous with the MIKC type) are considered the key regulatory genes of growth and development in plants [41]. In addition to the MADS domain, their amino acid sequence contains the I region, L (loop), K domain, and C-terminal domain [42]. Type II MADS-box genes underwent a slower birth-and-death evolution in angiosperms, resulting in higher rates of duplication and pseudogenization when compared to Type I genes [43]. Type II MADS-box genes have roles in the ABCDE flowering model [44,45]; these proteins usually function through tetramer formation (floral quartet model) that cause the promoter region containing *CArG*-*box* sites to loop, thus activating the transcription process [46].They also have roles in both gametophytic and sporophytic formation in non-seed plants, contributing to vegetative and reproductive structure development [42]. In seed plants, they are crucial for various aspects of sporophyte development, including floral organ specification [37]. The Type II genes are further subdivided into MIKC^C^ and MIKC* subgroups [37]. They are found in streptophytes with a K domain, but are absent in rhodophytes, glaucophytes, prasinodermophytes, and chlorophytes [47].

In this study, a comprehensive analysis of the MADS-box gene family in teak was performed. Utilizing the new chromosome-scale genome assembly of teak plant, 87 MADS-box genes were identified and classified into subfamilies. The amino acid sequences were analyzed for their conserved motifs and phylogenetic relationships with orthologous genes from other plant species. The chromosomal distribution of the identified MADS-box genes and gene structure of teak MADS-box genes were analyzed. In addition, expression profiles across different tissues were performed using publicly available transcriptomic data. Structures of key *T. grandis* MADS-box genes involved in the ABCDE flowering model were predicted, and the molecular dynamics and the stability of the protein–protein interaction (PPI) were analyzed. Finally, the putative functional roles of key MADS-box genes in developmental processes were discussed and suitable candidates for future functional studies were highlighted.

## 2. Materials and Methods

### 2.1. Identification of TgMADS Genes

The teak genome assembly and protein annotation (BioProject: PRJNA493753) were downloaded from publicly available GIGA databases [48]. Thereafter, hidden Markov model (HMM) profiles for the SRF-type MADS domain (PF00319) and MEF2 domain (PF09047) were obtained from the Pfam database, as described previously [49]. The retrieved HMM profiles were used as queries in HMMER searches (HMMER v3.0) against the *T. grandis* proteome dataset using the default parameters (Hits with e-values < 10^−3^ were considered significant hits (Supplementary Table S1). Full consensus sequences comprised HMMR3 score). The candidate TgMADS proteins were further analyzed using the Conserved Domain Database (CDD) and ScanProsite and SMART tools to confirm the presence of the MADS domain [50,51]. Redundant sequences were then removed, and the remaining genes were considered as putative teak MADS-box genes. Each Type II (MIKC) candidate gene missing the K domain was reannotated using the FGENESH suite [52,53], with the *TgMADS* genomic DNA sequences referenced against *S. indicum* genes. The TgMADS amino acid sequences were analyzed for their theoretical molecular weights and isoelectric points (pI) using the ProtParam tool [54].

### 2.2. TgMADS Genes Chromosomal Mapping and Gene Structure Analysis

The chromosomal positions of the *TgMADS* genes were obtained from genome annotation data and mapped onto the 18 pseudo-chromosomes of *T. grandis* using the TBtools software suite v2.323 [55]. Gene duplication events (tandem and segmental duplication) were inferred using the genomic position, gene order, and synteny-based evidence [56]. Tandem duplication events were identified based on strict physical adjacency (no intervening annotated genes were present between them). For segmental duplication, genome-wide protein similarity searches were performed using BLASTP, and reciprocal best-hit relationships (RHBs) were used to identify homologous gene anchors, and each *TgMADS* paralog supported by two or more conserved RBH flanking gene anchors (threshold set to >5) were classified as segmental duplicates. To examine the gene structure, exon–intron organization was visualized with the Gene Structure Display Server (GSDS 2.0) [57] by aligning the coding sequences of *TgMADS* genes with their corresponding genomic regions, based on the *T. grandis* GFF3 annotation file retrieved from the GIGA database [48].

Conserved motifs within TgMADS proteins were predicted using MEME (Multiple Expectation Maximization for Motif Elicitation [58]), with the motif widths ranging from 6 to 200 amino acids and the maximum number of motifs set to 20. The motifs were annotated by comparison against the Pfam and SMART databases.

### 2.3. TgMADS Proteins Phylogenetic Analysis

To assess the evolutionary relationships, the TgMADS Type I and Type II MADS-box proteins were aligned with the MADS-box proteins from *S. indicum, A. thaliana*, and *A. trichopoda* [59] using UGENE MUSCLE [60] and MEGA 12 software [61] with the following parameters: Gap Open: −2.9; Gap Extension: 0.0; Hydrophobicity Multiplier: 1.2; Cluster Method: UPGMA (for computationally less intensive establishment of initial branching order). Thereafter, the alignment was used to construct the maximum likelihood (ML) phylogenetic trees in MEGA X (1000 bootstraps; model: Jones–Taylor–Thornton (a gold-standard suitable for nuclear proteins); rates: uniform; gaps: use all sites replicates) that included *T. grandis*, *A. thaliana*, *S. indicum,* and *A. trichopoda* Type I and Type II MADS-box proteins. Using the same approach, circular and linear phylogenetic trees were constructed for all members of TgMADS protein family.

### 2.4. Expression Profiling of TgMADS Genes

Publicly available RNA-Seq datasets containing *T. grandis* RNA-Seq data from teak tissues (including the roots, seedlings, leaves, flowers, branches, and stems of 12- and 60-year-old specimens) were downloaded from the GIGA database (BioProject: PRJNA493753). Transcript abundances (TPM) were estimated by converting the Fragments Per Kilobase of transcript per million (FPKM) data to transcripts per million (TPM) using the formula: TPM = FPKM/[sum of all FPKM of a sample] × 10^6^. The differential expression patterns were visualized with heatmaps generated in Microsoft® Excel® for Microsoft 365 MSO (Version 2512 Build 16.0.19530.20184) 64-bit..

### 2.5. Protein Structure Predictions and Molecular Dynamics Simulations

The protein monomers’ and tetramers’ 3D structures were predicted using the amino acids sequences submission to Alphafold3 (AF3) server accessed on 16th January 2026 [62]. Molecular dynamics simulations (MDS) assays were performed using GROMACS 2026.0 [63]; the complexes were dissolved in TIP3P water dodecahedron and neutralized with 0.15 M NaCl at a constant pressure of 1 bar and temperature of 300 K. All the structures were visualized by BIOVIA Discovery Studio Visualizer v24.1.

## 3. Results

### 3.1. Identification, Genomic Distribution, and Gene Structure of TgMADS Gene Family

Eighty-seven non-redundant *MADS-box* genes were identified using the HMMER toolkit (HMMER) on the *T. grandis* proteome sequences (Table 1). These genes were designated *TgMADS1* to *TgMADS87* based on their chromosomal positions, and the presence of the MADS domain was confirmed using SMART [50] and PROSITE servers (accessed on 5 April 2025) [51]. Using this approach, a number of *TgMADS* genes (*TgMADS 4, 5, 22, 25, 27, 29, 36, 39, 45, 47, 48, 49, 51, 61, 62, 66, 71, 79, 84*) were identified as MADS-box genes. The other identified *TgMADS* genes were either lacking the K domain, contained odd domains (e.g., transmembrane domains), or the K domain was too short (e.g., only one of the three α-helices was present). This might have been due to the common limitations in the automated assembly and annotation pipelines in genomics studies. Therefore, further inspection of the genomic data was carried out to identify functional sites, followed by a genome annotation analysis using the FGENESH suite [52]. For this purpose, the *TgMADS* genomic DNA sequences were used in reference to the *S. indicum* genes to predict the correct gene structure, and the corrected TgMADS protein annotations contained both the M and K domains, while the odd domain exons were removed.

**Figure 1:**
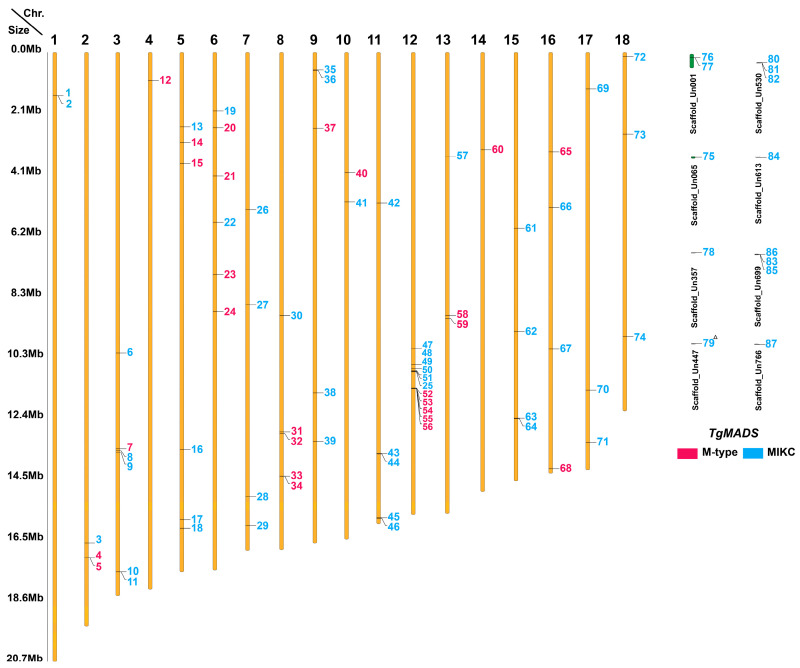
Chromosomal localization of the 87 *T. grandis MADS-box* genes. The number of each partial chromosome (pseudomolecule) is given under the lines; pink indicates M-type genes, and blue indicates MIKC type. The right side of each chromosome is related to the approximate physical location of each *MADS-box* gene. ^Δ^ Gene is partial.

Seventy-four *TgMADS* genes were physically mapped across the 18 pseudo-chromosomes of the *T. grandis* draft genome, with 13 *TgMADS* genes located on unanchored scaffolds (Figure 1). The chromosomal distribution of *TgMADS* genes was uneven, with chromosome 12 harboring the highest number of genes (11; 12.6%), whereas chromosomes 4 and 14 each contained only a single gene. Several *TgMADS* members were organized into localized clusters containing 2–5 consecutive genes, suggesting potential tandem and segmental duplication events. For the tandem duplications, 10 events were identified across the *T. grandis* genome that were distributed among five pseudo-chromosomes, with one pair on pseudo-chromosome 8, one pair on pseudo-chromosome 9, two pairs on pseudo-chromosome 11, five pairs on pseudo-chromosome 12, and one pair on pseudo-chromosome 15, and two events in two unanchored scaffolds (Un530 and Un699) (Supplementary Table S2: Tandemly repeated genes). Interestingly, pseudo-chromosome 12 harbored the highest number of tandemly duplicated *TgMADS* genes (five pairs), with four consecutive tandem pairs forming a localized gene cluster (*TgMADS 50*–*56*), suggesting a tandem duplication hotspot of *TgMADS* genes on this pseudo-chromosome. For the segmental duplication events, a total of 10 *TgMADS* gene pairs were identified using a MicroSynteny analysis with anchor support values ranging from 6 to 17, indicating a conservation between the duplicated loci (Supplementary Table S3: Segmental MicroSynteny analysis results). The segmental duplication events were also detected within the same chromosomes (intra) and between different chromosomes (inter). The intra-chromosomal segmental duplications (three events) were observed on chromosomes 3, 5, and 13, where the duplicated *TgMADS* gene pairs were separated by large genomic distances within the same chromosome. In contrast, the inter-chromosomal segmental duplications (seven events) were distributed across different chromosomes, including duplications between chromosomes 3 and 18, 7 and 9, and 11 and 15, and between anchored chromosomes and unplaced scaffolds. On the other hand, four *TgMADS* genes found in tandem duplication events (two events) were also identified as segmental duplicates (one event), with the paralogs located on distant chromosomal regions. In addition to the chromosome-based genes, those located on unanchored scaffolds were also examined, revealing a total of 10 putative paralogous gene pairs, 6 of the MIKC type (*TgMADS* {*1, 2*}, {*17, 18*}, {*76, 77*}) and four of the M type (*TgMADS* {*4, 5*}, {*52, 85*}). These duplication patterns likely contributed to the evolutionary expansion and functional diversification of the *MADS-box* gene family in *T. grandis*.

### 3.2. Phylogenetic Analysis of TgMADS Gene Family

**Figure 2:**
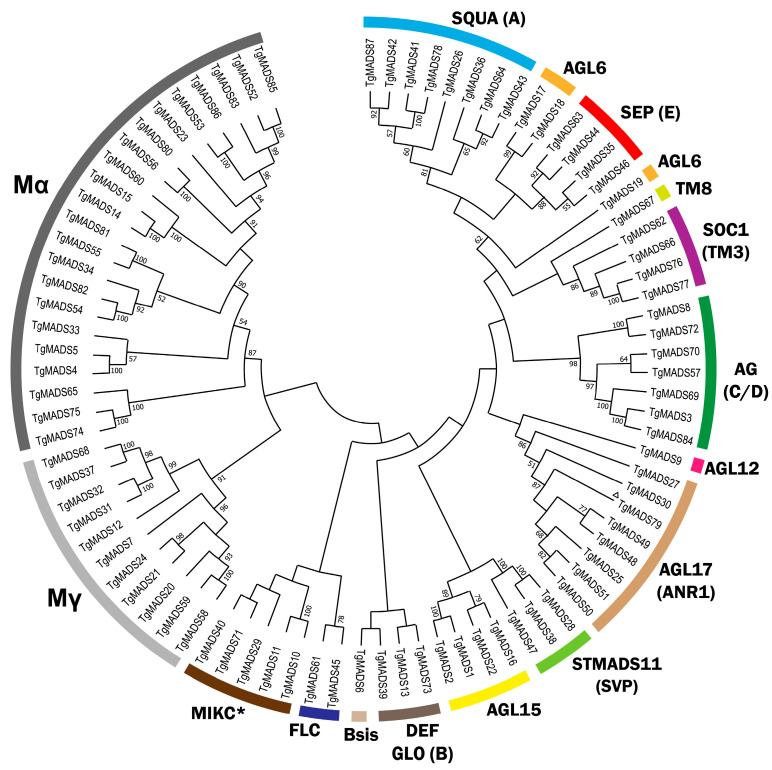
Maximum likelihood phylogenetic tree of TgMADS proteins. Type I subgroups (Mα and Mγ) are shown; Mβ subgroup is not present. Type II proteins are classified into MIKCc subfamilies, including SQUA (A), DEF/GLO (B), AG (C/D), SEP (E), AGL6, AGL12, AGL15, AGL17 (ANR1), Bsis, TM3/SOC1, STMADS11 (SVP), FLC, and TM8. ^Δ^ Gene is partial.

**Figure 3:**
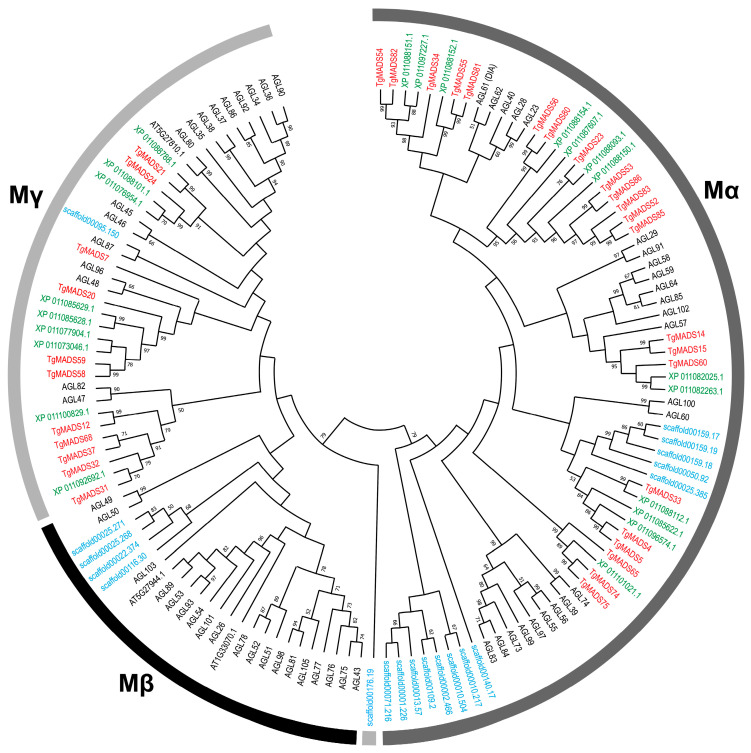
Maximum likelihood phylogenetic tree of Type I MADS-box proteins from *T. grandis*, *A. thaliana*, *S. indicum*, and *A. trichopoda*. Proteins from each species are distinguished by color: *T. grandis* (red), *S. indicum* (green), *A. trichopoda* (blue), and *A. thaliana* (black).

**Figure 4:**
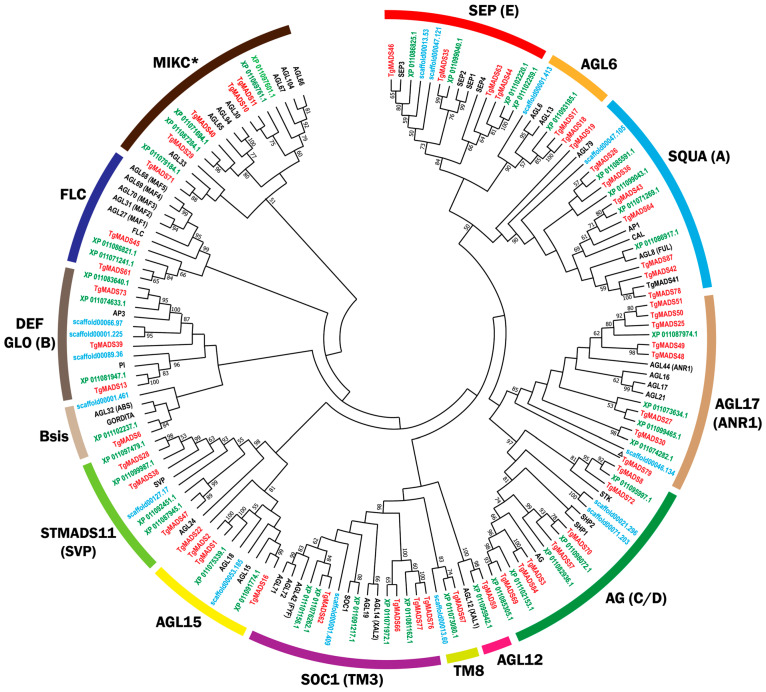
Maximum likelihood phylogenetic tree of Type II MADS-box proteins from *T. grandis*, *A. thaliana*, *S. indicum*, and *A. trichopoda*. Proteins from each species are indicated by colored circles: *T. grandis* (red), *S. indicum* (green), *A. trichopoda* (blue), and *A. thaliana* (black). ^Δ^ Gene is partial.

To classify the TgMADS proteins accurately, three maximum likelihood phylogenetic trees were constructed. The first phylogenetic tree included all the identified TgMADS proteins (Figure 2), whereas the other two illustrated the evolutionary relationships within the Type I (Figure 3) and Type II (Figure 4) subfamilies. Each tree was generated using full-length MADS-box amino acid sequences from *T. grandis* in combination with representative MADS-box proteins from *A. thaliana*, *S. indicum*, and *A. trichopoda*. In addition, the TgMADS proteins were assigned to functional groups following the classifications previously described for *A. thaliana* and *S. indicum* [32,33]. This phylogenetic analysis identified 54 Type II and 33 Type I genes, which were further subdivided into four well-defined clades: MIKC^C^ and MIKC* (Type II) and Mα type and Mγ type (Type I). Interestingly, the Mβ-type subgroup of Type I MADS-box genes was absent from the *T. grandis* genome (Figure 3), which is in agreement with previous analysis of *S. indicum* [64] and *Callicarpa americana* [65]. The phylogenetic analysis with Arabidopsis, Sesame, and Amborella genes showed that the *T. grandis* MIKC^C^ genes were grouped into well-defined subfamilies, such as AGAMOUS, APETALA1, SEPALLATA, and PISTILLATA, indicating conservation of floral identity gene lineages among the studied species.

### 3.3. Conserved Motif Distribution and Gene Structure Analysis of TgMADS Genes

**Figure 5:**
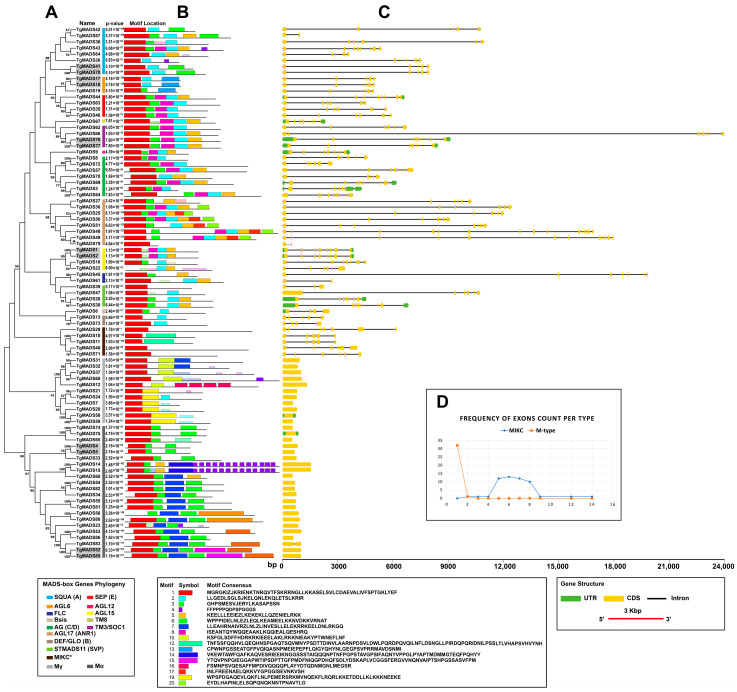
Gene structure and conserved motif analysis of TgMADS proteins. (**A**) Phylogenetic relationships among TgMADS proteins. (**B**) Conserved motif composition: Each motif is shown as numbered, colored box, with box length reflecting motif length. (**C**) Exon–intron structures of *TgMADS* genes (exons in yellow, introns as solid lines, and untranslated regions in green). (**D**) Exon frequency distribution by MADS-box type (M type in orange, MIKC in blue). ^Δ^ Gene is partial.

For the gene structure analysis, exon numbers of *TgMADS* genes varied from 1 to 14 exons, with the Type II *MADS-box* genes (2–14) showing noticeably higher exon numbers than the Type I genes (1–2). *TgMADS79* was excluded from the above calculations as the gene was not complete due to short contig assembly. The exon numbers were generally conserved within each subgroup, except for a few minor exceptions. The exon–intron organization of the coding sequences of each *TgMADS* gene are shown in Figure 5—right. The exon distribution exhibited a distinct bimodal trend, where all the Type II (MIKC) *TgMADS* genes consistently contained an average of six exons. The lowest exon number was 2 exons (*TgMADS11* in the SQUA subgroup), and the highest numbers were 12 and 14 exons (*TgMADS49* and *TgMADS48*, respectively, in the AGL17 subgroup). In contrast, the Type I (M type) *TgMADS* genes typically contained a single exon and lacked introns, except for the Mα subgroup, where *TgMADS83* contained two exons. Additionally, a few members of the MIKC group exhibited notably long introns—exceeding 10 kb—compared with the rest of the *TgMADS* genes.

Most of TgMADS proteins showed alkaline isoelectric points, with 59 proteins exhibiting pI values above 7.5, whereas only 6 fell in the neutral range (6.5–7.5), and 27 were considered acidic with pI values below 6.5. Interestingly, all members of the MIKC* subgroup were acidic, averaging a pI of 6.29, while the M-type proteins averaged 7.43 and the MIKC^C^ group averaged 8.21, reflecting a basic character. The predicted molecular masses also varied among the classes: the MIKC^C^ proteins had a mean mass of approximately 25.7 kDa, the MIKC* proteins about 29.7 kDa, and the M-type proteins around 30.2 kDa. Such variation in the pI and molecular weight among TgMADS proteins has also been observed in other species, where subsets of the family separate into acidic, neutral, and basic.

The sequence features and motif architecture of all the TgMADS proteins were analyzed using the MEME suite, which identified and visualized the conserved motif patterns and their structural variations across the different TgMADS groups (Figure 5, left panel). As expected, Motif 1 corresponds to the canonical MADS domain, comprising 57 amino acids, which is responsible for DNA binding. Motif 3 represents the I domain, while motifs 8, 2, and 5 collectively define the highly conserved K domain, subdivided into K1, K2, and K3 segments with a total length of 87 amino acids (29 + 29 + 29). These motifs, identified through the MEME analysis, were found either fully or partially across all the MIKC-type TgMADS proteins. In several TgMADS proteins, however, certain M and K domains were not detected by the MEME analysis and, therefore, a secondary validation was performed using the SMART, PROSITE, and MotifFinder tools, which confirmed their presence. In general, the same subgroups of TgMADS proteins had similar motif structures, indicating they might have conserved and similar functions. Conversely, the observed variation in the motif structure and distribution among the TgMADS members implies divergent functional specialization across different organs of *T. grandis*.

### 3.4. Expression Profile of TgMADS Genes

**Figure 6:**
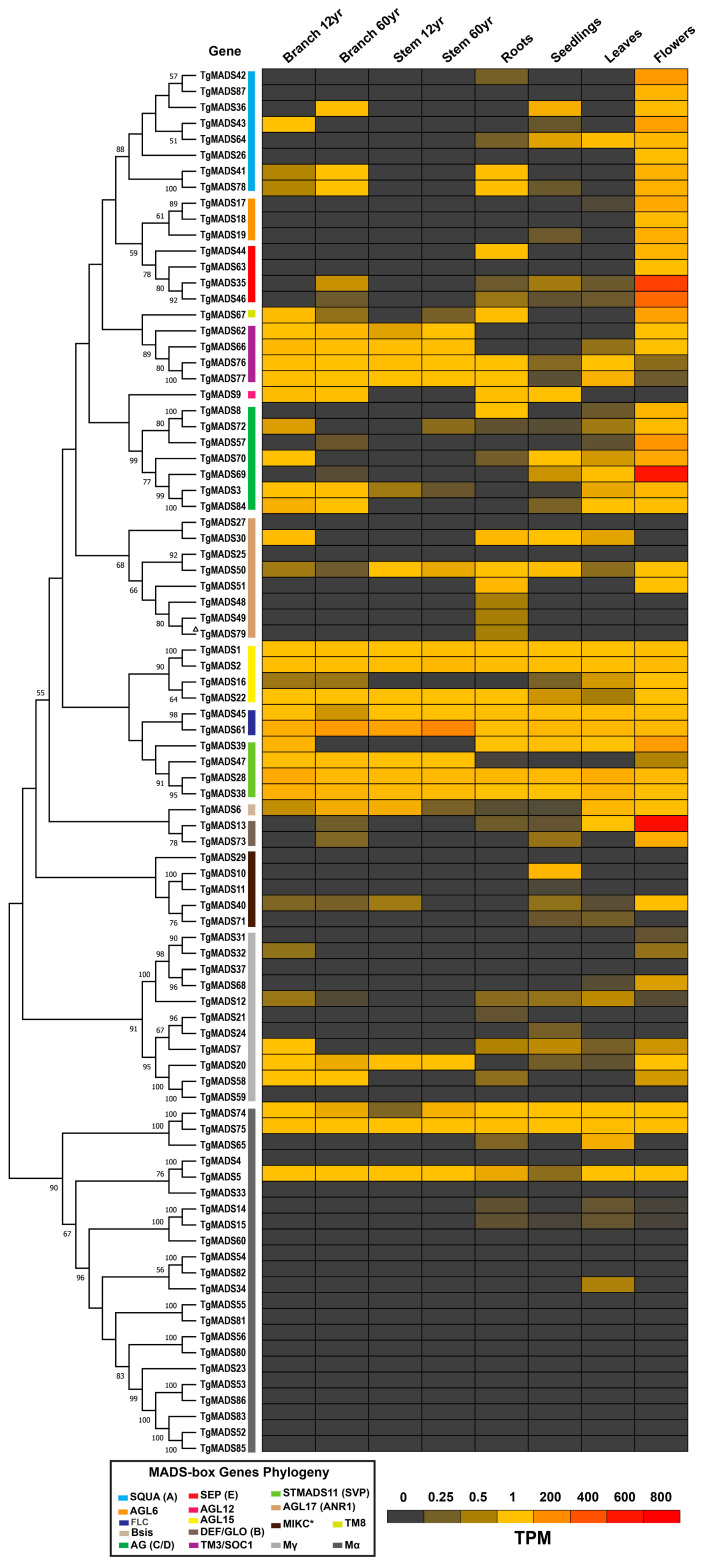
Heatmap of *TgMADS* genes expression levels (TPM) across root, seedling, leaf, flower, stem, and branch tissues from 12- and 16-year-old specimens. The phylogenetic tree is shown on the left, and the subgroup classification of *TgMADS* genes is indicated in the box at the lower left. ^Δ^ Gene is partial.

The expression patterns of the 87 *TgMADS* genes were used to construct a heat map after converting the Fragments Per Kilobase of transcript per million mapped reads (FPKM) values into transcripts per million (TPM) values [12]. The gene expression patterns were analyzed across multiple tissues, including the roots, seedlings, leaves, flowers, stems, and branches of 12- and 16-year-old trees (Figure 6). Overall, the *TgMADS* genes were actively expressed in all the examined tissues, underscoring their essential roles in key developmental and physiological processes.

In the floral organs, the Type II (MIKC) *TgMADS* genes displayed the highest expression levels, with several members showing exclusive expression in floral tissues. This observation is consistent with their known function as core regulators of the florogenesis pathway. The results further support the involvement of *TgMADS* genes in the ABCDE model of flower development and confirm the conservation of this regulatory framework in *T. grandis*. Within the MIKC subgroup, members of the DEF/GLO (B) class—particularly *TgMADS13*—showed the highest expression in flower tissues, followed by *TgMADS69* from the AG (C/D) subgroup and *TgMADS35* and *TgMADS46* from the SEP (E) subgroup. In contrast, most Type I *TgMADS* genes exhibited low or undetectable expression in the sampled tissues. Among the Type I *MADS-box* genes, *TgMADS74, TgMADS75*, and *TgMADS5* (members of the Mα subgroup) were expressed across all the examined tissues, whereas *TgMADS20* from the Mγ subgroup showed a similar expression pattern except in the roots. A few additional Type I genes exhibited weak expression in the floral tissues.

### 3.5. Structural Prediction and Protein–Protein Interaction Modeling

**Figure 7:**
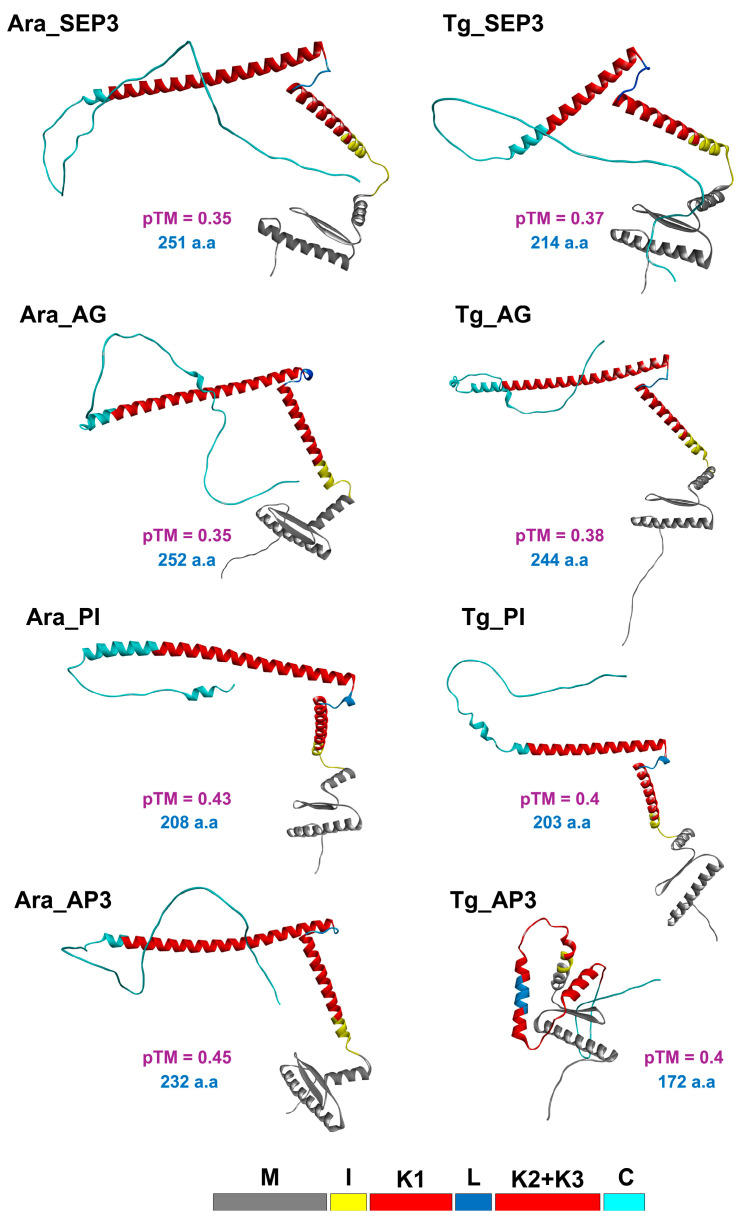
Structure prediction of selected *T. grandis* and *A. thaliana* MADS-box genes involved in the ABCDE flowering model. Predicted template modeling (pTM) scores in purple, amino acids length in blue.

The structures of SEP3, AG, PI, and AP3 orthologs in the *T. grandis (Tg)* and *A. thaliana (Ara)* MADS-box genes involved in the ABCDE flowering model [44,45] were predicted via AF3 (Figure 7). All protein orthologs showed the key M, I, K1, Loop, K2, K3, and C domains in the Type II MIKC^C^ subfamily [42,66]. It is worth noting that the TgMADS proteins were shorter than the *A. thaliana* orthologs; mainly the I and K domains were shorter, while the M domain was conserved in length. All the predicted structures were overall similar except Tg-AP3, which had a very short overall length of 172 amino acids (a.a) compared to the 232 in Ara-AP3; this resulted in a very short I domain and K2-K3 domains.

**Figure 8:**
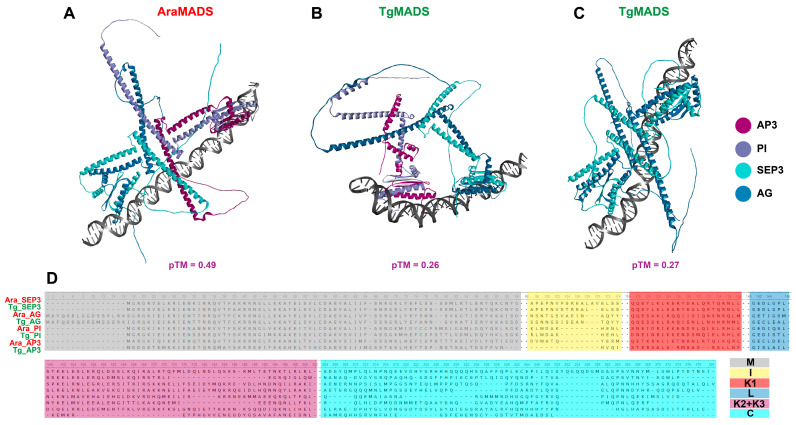
Tetramer formation and DNA binding simulation of *T. grandis* and *A. thaliana* (SEP3, AG, PI, and AP3) MADS-box proteins. (**A**) *A. thaliana* (SEP3, AG, PI, and AP3) tetramer formation; (**B**) *T. grandis* (Tg-SEP3, Tg-AG, Tg-PI, and Tg-AP3) tetramer formation; (**C**) *T. grandis* (Tg-SEP3, Tg-AG, Tg-SEP3, and Tg-AG) tetramer formation; (**D**) multiple sequence alignment of *A. thaliana* and *T. grandis* (SEP3, AG, PI, and AP3) MADS-box proteins orthologs. Predicted template modeling (pTM) scores in purple.

To verify the potential of these proteins to form floral quartets, a 118 nucleotides sequence containing two *CArG* sites toward its ends was used to compare the tetramer formation of one of the quartets in the stamens that consisted of members of class E (SEP3), class C (AG), and class B (PI, AP3) (Figure 8).

The M domains were always able to dimerize and bind to the DNA as predicted. The A. thaliana proteins were able to form tetramers mainly through coiled-coil interactions between the K2 and K3 domains, as reported in wet lab assays [67]. The Tg-SEP3 (TgMADS46) and Tg-AG (TgMADS69) had similar interaction patterns as the Ara-SEP3 and Ara-AG proteins, but Tg-PI (TgMADS13) and Tg-AP3 (TgMADS39) had different interaction patterns compared to Ara-PI and Ara-AP3. This was mainly due to the odd shaped, shorter Tg-AP3 protein. Nonetheless, they were able to form tetramers. To investigate the ability of T. grandis proteins to form similar structured tetramers as the A. thaliana orthologs, another quartet (SEP3, AG, SEP3, and AG) found in carpels was simulated (Figure 8C). This quartet had a very similar tetramer standard formation, as seen in the A. thalian orthologs, were the K2 and K3 domains are involved in the PPI along the stretched α-helices.

**Figure 9:**
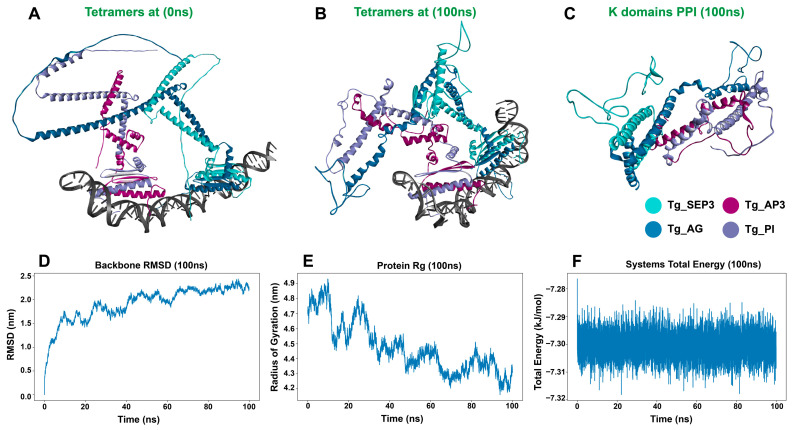
*T. grandis* floral quartet (Tg-SEP3, Tg-AG, Tg-PI, and Tg-AP3) tetramer molecular dynamic simulations. (**A**) The AF3 predicted structures at 0 ns; (**B**) simulated tetramer at 100 ns; (**C**) top view of the K domains interactions; (**D**) the tetramer backbone RMSD calculation over 100 ns; (**E**) the tetramer radius of gyration calculation over 100 ns; (**F**) the system total energy calculation over 100 ns.

To confirm the stability of the predicted *T. grandis* tetramers, and the possible dynamics of the odd shortened Tg_AP3 protein, an MDS was carried out for 100 ns (Figure 9).

Overall, the tetramer remained stable and bound to the promoter DNA over the 100 ns simulation time. The K2 and K3 domains remained the key domains involved in tetramerization, but the Tg-AP3 K3 domain interacted with the Tg-PI K3 domain in a perpendicular angle rather than the expected parallel geometry, and the Tg-AP3 domain seemed to play a role in the PPI with Tg-PI as well.

## 4. Discussion

In plants, the *MADS-box* gene family exhibits considerable variation in the numbers and types of its members, with the Type I (M type) genes showing particularly dynamic evolutionary patterns, with faster birth-and-death evolution compared to the Type II genes [37]. For instance, green algae (chlorophytes) typically lack or have few MIKC (Type II) MADS-box genes, suggesting major differences in MADS-box gene evolution between different plant lineages [37]. In contrast, angiosperms have experienced significant expansions of specific Type I subclades, particularly those associated with reproductive development, with angiosperm-specific Type I clades (Mγ and Mα) originating at the base of flowering plants and undergoing subsequent duplications and neofunctionalization [68]. For instance, Arabidopsis contains 64 functional Type I genes while rice has only 24 functional Type I genes, demonstrating the rapid turnover characteristic of this gene family [69]; this is reflected in the lower bootstrap values calculated in the phylogenetic tree. Type I genes often lack the conserved K domain found in Type II genes and are under weaker purifying selection. This leads to higher sequence divergence, which can cause long branch attraction and lower bootstrap support. Furthermore, Type I (M type) MADS-box genes are found in a limited number of organisms, while some lack them entirely; examples include *Saccharum officinarum* (sugarcane); *Marchantia polymorpha*; and the algal species *Klebsormidium flaccidum*, *Dunaliella salina*, and *Chlorella variabilis*. Among land plants, *M. polymorpha* contains only two Type II genes, the gymnosperm *Picea abies* has three, while the carrot (*Daucus carota*) has five. In contrast, other angiosperms exhibit a lineage-specific expansion of both groups, with *Camelina sativa* harboring the largest complement of Type I genes (271), whereas *Glycine max* (soybean) contains the highest number of Type II genes (209) [53,59]. Collectively, these evolutionary patterns highlight the variability in *MADS-box* gene families across the plant kingdom and reflect their central involvement in diverse developmental pathways and their rapid evolution under different selective pressures.

Within the Lamiaceae family, the number of Type I *MADS-box* genes in *T. grandis* (33) closely matches those in *S. indicum* (31) and *C. americana* (32), though it remains lower than in *Ocimum tenuiflorum* (42). For Type II genes, *T. grandis* (54) has a higher count than *O. tenuiflorum* (43) and *C. americana* (46), yet fewer than *S. indicum* (62). The genome size of *T. grandis* is 338 Mb [12], nearly identical to *S. indicum* (337 Mb) [64], and smaller than *O. tenuiflorum* at 612 Mb [70]. It is also notably smaller than the soybean genome (1115 Mb) [71], which contains 269 *MADS-box* genes, and is smaller than *Camelina sativa* at 785 Mb [72], which carries 384 *MADS-box* genes. The low number of *MAD-box* genes observed in certain Lamiaceae species may be associated with their smaller genome sizes and possible genome size reduction following duplication events. Such events are known to play a key role in the expansion and diversification of gene families in plants [64,68,73,74]. This is further supported by the presence of tandem and segmental duplicates, which indicate the contribution of large-scale duplication processes in *TgMADS* family expansion and evolution, and provide evidence for the non-random chromosomal distribution. It is worth mentioning that the observed enrichment of *TgMADS* genes on pseudo-chromosome 12 is mainly attributed to a localized tandem duplication event (four gene pairs in tandem), whereas the segmental duplication analysis indicates a broad distribution of *TgMADS* genes across the genome, consistent with the distinct evolutionary mechanisms underlying local gene clustering versus large-scale duplication [75]. Furthermore, *MADS-Box* gene clustering likely originated through duplication followed by functional divergence [74,76]; such phenomenon has also been reported in other transcription factor families, such as HOX genes [77]. The higher exon counts observed in *Type II (MIKC)* genes (2–14) compared with *Type I* genes (1–2) are consistent with previous findings for *S. indicum*, *A. thaliana*, *O. sative*, and *G. max* [64,71,73]. This pattern reflects the greater structural complexity and broader functional diversity typically associated with Type II genes relative to Type I [37,44,76,78]. Collectively, these evolutionary patterns highlight the variability in *MADS-box* gene families across the plant kingdom and reflect their central involvement in diverse developmental pathways and their rapid evolution under different selective pressures.

The absence of the Mβ subgroup in members of the Lamiaceae family *T. grandis*, *S. indicum*, and *C. americana* [65] points to a targeted contraction of this Type I MADS-box clade. Type I MADS-box genes are characterized by a rapid birth-and-death evolution; thus, the loss of Mβ likely reflects a period of intensive genome fractionation following ancestral polyploidy events in the Lamiales. Furthermore, given the specialized role of Mβ genes in endosperm development [38,73], its absence may be linked to the diverse and often specialized seed developmental strategies observed across this order. The loss of Mβ may indicate a functional shift where its ancestral role in seed nourishment was either lost or reassigned to expanded Mα and Mγ paralogs, coinciding with the evolution of cellular endosperm development and haustorial endosperm structures characteristic of this order [79,80]. This suggests that while MIKC^C^ genes remain conserved as a ‘core toolkit,’ the Type I Mβ lineage might be prone to lineage-specific extinction when its functions become redundant or the underlying developmental processes shift. Mβ-type genes have also been reported missing in rice and other monocots [73], which supports the view that this subgroup likely evolved as a lineage-specific clade.

*TgMADS67*, a *TM8* gene ortholog, is found in *S. indicum*, *S. lycopersicum*, and *Nicotiana benthamiana*, yet it is absent in *A. thaliana*. The expression pattern across multiple tissue types, combined with the absence of a well-defined phenotype when *TM8* is deleted or overexpressed, has made it challenging to determine their precise role. In *N. benthamiana*, *TM8* represses the microRNA miR172 together with an *SVP-type* gene [81]. In *S. lycopersicum*, the overexpression or repression of *TM8* changes sexual organs viability, shape, and some floral identity gene expression [82]. Additional molecular and spatiotemporal expression profile studies on the *TgMADS67* ortholog in *T. grandis* could help clarify the biological function of this still poorly understood gene.

Overall, at least one *TgMADS* gene was actively expressed in every tissue examined, emphasizing the broad functional range and biological significance of MADs-box gene family in *T. grandis*. The expression was generally higher among the Type II *TgMADS* genes compared to Type I, a pattern consistent with their structural complexity and functional diversification [37,44,76,78]. In the floral tissues, *TgMADS13*, the ortholog of Arabidopsis *PISTILLATA* (*PI*), displayed the strongest expression, together with *TgMADS35*, the ortholog of *SEPALLATA2* (*SEP2*); *TgMADS46,* the ortholog of *SEPALLATA3* (*SEP3*); and *TgMADS69,* the ortholog of *AGAMOUS* (*AG*). This pattern reflects the pivotal regulatory functions of *PI*, *AG*, and *SEP* genes and their paralogs during floral organ development [83]. Two other genes, *TgMADS45* and *TgMADS61*, corresponding to Arabidopsis *MAF3* and *MAF2* of the *FLC* subgroup, were also expressed in the flowers. However, their transcript levels do not appear to align with the repression of flowering observed in Arabidopsis [84]. Interestingly, no *FLC* ortholog was detected in *T. grandis*, which may suggest modifications in the vernalization response and a potential role of the *MAF2* and *MAF3* paralogs, which in *A. thaliana* act as repressors to prevent premature flowering during transient cold exposure [85]. Similar losses of *FLC* have been reported in other taxa, including orchids [86], where *SVP* and *AGL25* orthologs likely act as floral repressors. Furthermore, *TgMADS40*, an ortholog of Arabidopsis *AGL65*, which belongs to the MIKC* subgroup, was expressed in floral tissues, implying a possible conserved role in male gametophyte development, particularly in the later stages of pollen maturation and pollen tube growth [76,87,88]. Some MIKC-type genes in *T. grandis* showed expression in the roots, stems, and leaves; this agrees with observations in *A. thaliana*, where several *MADS-box* genes function beyond flower development. For example, *SVP* and *SOC1* contribute to drought tolerance, while *ANR1* and *AGL21* regulate lateral root formation. Consistently, the corresponding orthologs *TgMADS28*, *TgMADS38*, *TgMADS49*, and *TgMADS79* are expressed in *T. grandis* roots.

In Arabidopsis, most of Type I *MADS-box* genes were found to be expressed at a low level, and their function is not as well understood as the Type II *MADS-box* genes. The expression of the following Type I genes in flower bud tissues—*TgMADS5*, *74* and *75* orthologs of Arabidopsis *AGL62*; *TgMADS68*, *32* orthologs of Arabidopsis *AGL47*; *TgMADS58,* ortholog of Arabidopsis *AGL45*; *TgMADS20*, ortholog of Arabidopsis *AGL35*; and *TgMADS7*, ortholog of Arabidopsis *AGL80*—suggests their potential involvement in floral development. These expression patterns are consistent with earlier reports showing that certain Type I *MADS-box* genes participate in specific reproductive and developmental processes in *A. thaliana* [38,73]. Interestingly, several genes showed no detectable expression in any *T. grandis* tissue, and such absence may indicate that some *MADS-box* genes might be conditionally expressed under particular environmental factors or abiotic stresses, such as temperature, salinity, drought, or mechanical injury [89,90]. Another possible explanation is that some of these genes represent pseudogenes with no active function or redundant duplicates undergoing neofunctionalization. The presence of two or more *A. thaliana MADS-box* orthologs in *T. grandis* indicate functional redundancy, or the gaining of new regulatory roles. Such genes might also display differential expression patterns in response to environmental cues, allowing fine-tuned transcriptional control within *T. grandis*. Genomic studies of *T. grandis* have identified at least one whole-genome duplication event [12], consistent with recent findings of gene duplications reported in mint plants [91]. Indeed, such events are known to play a major role in the expansion and diversification of the MADS-box gene family [68].

The ABCDE flowering model seems to be conserved in *T. grandis*. The floral quartets—Tg-SEP3, Tg-AG, Tg-AP3, and Tg-PI found in the stamens, and Tg-SEP3, Tg-AG, Tg-SEP3, and Tg-AG found in the carpels—were able to form tetramers and bind to the DNA promoter sequence. Interestingly, the *T. grandis* orthologs were overall shorter than the *A. thaliana* orthologs. In particular, the Tg-AP3 protein was 60 amino acids shorter than Ara-AP3, with noticeably shortened I and K domains. Nonetheless, the floral quartet formed during simulation seemed to be stable, although it had a peculiar PPI pattern, where the α-helix of the K3 domain of Tg-AP3 was bound to the α-helix of the K3 domain of Tg-PI at a perpendicular angle, rather than the expected parallel coiled-coil structure [66,67]. In addition, the C domain of Tg-AP3 seems to have a role in the PPI as well. These might be adaptations to stabilize the complex due to the shortened K domains, but future wet lab PPI assays will be essential to confirm these odd interaction patterns, and their relevance to the overall protein network dynamics. In addition, future MDS assays of the complex for longer durations with modified promoter lengths might also help shed light on this PPI stability. These observations on possible alternative modes of PPI in the floral quartets might help further our understanding of the complexity of the overall PPI dynamics, and it might shed more light on the role of the variable unstructured C domain in the PPI in this key transcription factors family.

## 5. Conclusions

Using the latest *T. grandis* genome assembly and RNA-Seq datasets, a total of 87 *TgMADS* genes were identified through multiple bioinformatics analyses. These genes were categorized into Type I (Mα and Mγ) and Type II (MIKC* and MIKC^C^) clades based on their phylogenetic relationships and protein structural features. Mβ-type genes were not detected, consistent with their absence in other Lamiales species. The gene structure analysis revealed that the Type II genes contain more exons than the Type I genes. The expression profiling across eight tissues suggests that functions related to abiotic stress responses and the ABCDE floral regulatory model might be at least partially conserved in *T. grandis*. The variation in expression among some *TgMADS* genes suggests possible functional diversification or redundancy. The simulation of tetramers formation in the quartet model of key *T. grandis* orthologs revealed a stable complex formation that can bind to the DNA promoter sequence. Tg-AP3 had a significant reduction in protein length that might have resulted in an odd PPI, but further in silico and wet lab analyses are needed to confirm these peculiar dynamics. Our findings provide a foundation for future studies, including protein–protein interaction experiments, to clarify the regulatory functions of *TgMADS* genes, and to confirm the predicted PPI and the protein networks dynamics.

## Figures and Tables

**Table 1:** Detailed information for the *T. grandis MADS-box* gene family.

Gene Name	Gene ID	Chr	Exons	Chain	Length (aa)	PI	MW (kDa)	Group	Ortholog	E-Value	Subgroup
*TgMADS01*	*Tg01g02120.t1*	1	8	**−**	245	7.793	27,777.64	MIKC	AGL18	3 × 10^−49^	AGL15
*TgMADS02*	*Tg01g02320.t1*	1	8	**−**	245	7.793	27,777.64	MIKC	AGL18	3 × 10^−49^	AGL15
*TgMADS03*	*Tg02g16980.t1*	2	7	**+**	180	9.556	20,710.30	MIKC	AG	5 × 10^−89^	AG (C/D)
*TgMADS04*	*Tg02g17630.t1* *	2	1	**+**	217	6.083	24,501.89	M type	AGL62	3 × 10^−27^	α
*TgMADS05*	*Tg02g17650.t1* *	2	1	**+**	217	6.083	24,501.89	M type	AGL62	3 × 10^−27^	α
*TgMADS06*	*Tg03g08370.t1*	3	4	**+**	266	7.841	31,046.34	MIKC	ABS	2 × 10^−44^	Bsis
*TgMADS07*	*Tg03g12830.t1*	3	1	**−**	182	9.241	20,526.35	M type	AGL80	1 × 10^−20^	γ
*TgMADS08*	*Tg03g12970.t1*	3	6	**−**	212	9.548	24,662.10	MIKC	STK	4 × 10^−96^	AG (C/D)
*TgMADS09*	*Tg03g13060.t1*	3	7	**+**	215	8.2	24,680.66	MIKC	AGL12	9 × 10^−62^	AGL12
*TgMADS10*	*Tg03g18430.t1*	3	5	**−**	232	7.72	26,277.56	MIKC	AGL104	8 × 10^−32^	MIKC*
*TgMADS11*	*Tg03g18920.t1*	3	5	**+**	225	6.654	25,350.35	MIKC	AGL66	2 × 10^−32^	MIKC*
*TgMADS12*	*Tg04g01300.t1*	4	1	**−**	441	6.118	50,996.38	M type	AGL47	1 × 10^−18^	γ
*TgMADS13*	*Tg05g03610.t1*	5	6	**−**	203	6.329	23,680.87	MIKC	PI	8 × 10^−80^	DEF/GLO (B)
*TgMADS14*	*Tg05g04300.t1*	5	1	**−**	512	5.19	54,680.51	M type	AGL62	4 × 10^−26^	α
*TgMADS15*	*Tg05g05250.t1*	5	1	**+**	512	5.133	54,695.52	M type	AGL62	3 × 10^−26^	α
*TgMADS16*	*Tg05g13890.t1*	5	7	**+**	242	7.263	27,155.79	MIKC	AGL15	5 × 10^−48^	AGL15
*TgMADS17*	*Tg05g16950.t1*	5	5	**+**	189	8.515	21,175.17	MIKC	AGL6	4 × 10^−65^	AGL6
*TgMADS18*	*Tg05g17300.t1*	5	5	**+**	189	8.515	21,175.17	MIKC	AGL6	4 × 10^−65^	AGL6
*TgMADS19*	*Tg06g03090.t1*	6	5	**−**	178	5.736	19,809.03	MIKC	AGL6	9 × 10^−41^	AGL6
*TgMADS20*	*Tg06g03840.t1*	6	1	**−**	262	9.082	30,062.66	M type	AGL35	1 × 10^−19^	γ
*TgMADS21*	*Tg06g05880.t1*	6	1	**−**	257	8.127	28,493.16	M type	AGL80	7 × 10^−49^	γ
*TgMADS22*	*Tg06g08110.t1* *	6	7	**−**	288	6.323	32,718.19	MIKC	AGL15	6 × 10^−35^	AGL15
*TgMADS23*	*Tg06g09940.t1*	6	1	**−**	280	9.367	30,791.43	M type	AGL61	5 × 10^−37^	α
*TgMADS24*	*Tg06g11190.t1*	6	1	**−**	255	8.754	27,856.53	M type	AGL80	2 × 10^−59^	γ
*TgMADS25*	*Tg12gnew.t1* *	12	7	**+**	228	9.272	26,076.47	MIKC	AGL21	3 × 10^−86^	AGL17 (ANR1)
*TgMADS26*	*Tg07g06420.t1*	7	5	**+**	184	9.059	20,794.48	MIKC	FUL	3 × 10^−51^	SQUA (A)
*TgMADS27*	*Tg07g08930.t1* *	7	6	**−**	198	9.106	22,548.31	MIKC	AGL21	4 × 10^−65^	AGL17 (ANR1)
*TgMADS28*	*Tg07g13640.t1*	7	8	**−**	227	5.779	25,708.82	MIKC	SVP	1 × 10^−10^6	STMADS11 (SVP)
*TgMADS29*	*Tg07g14620.t1* *	7	8	**+**	326	6.098	37,662.41	MIKC	AGL67	5 × 10^−19^	MIKC *
*TgMADS30*	*Tg08g10570.t1*	8	6	**−**	222	8.635	25,471.88	MIKC	AGL16	1 × 10^−90^	AGL17 (ANR1)
*TgMADS31*	*Tg08g12890.t1*	8	1	**+**	305	8.086	34,967.83	M type	AGL47	5 × 10^−24^	γ
*TgMADS32*	*Tg08g12940.t1*	8	1	**+**	269	6.176	30,947.26	M type	AGL47	1 × 10^−25^	γ
*TgMADS33*	*Tg08g14560.t1*	8	1	**−**	249	5.23	27,010.24	M type	AGL62	3 × 10^−29^	α
*TgMADS34*	*Tg08g14570.t1*	8	1	**−**	248	7.467	28,084.16	M type	AGL62	3 × 10^−50^	α
*TgMADS35*	*Tg09g00890.t1*	9	6	**+**	218	7.807	24,813.87	MIKC	SEP2	1 × 10^−101^	SEP (E)
*TgMADS36*	*Tg09g00900.t1* *	9	6	**+**	220	9.438	25,887.28	MIKC	FUL	1 × 10^−60^	SQUA (A)
*TgMADS37*	*Tg09g02970.t1*	9	1	**−**	335	8.377	38,451.84	M type	AGL82	4 × 10^−20^	γ
*TgMADS38*	*Tg09g10160.t1*	9	8	**−**	228	6.521	25,795.07	MIKC	SVP	1 × 10^−11^3	STMADS11 (SVP)
*TgMADS39*	*Tg09g12140.t1* *	9	3	**−**	172	6.055	19,369.54	MIKC	AP3	2 × 10^−21^	DEF/GLO (B)
*TgMADS40*	*Tg10g05610.t1*	10	6	**+**	320	5.816	36,419.86	MIKC	AGL65	5 × 10^−44^	MIKC *
*TgMADS41*	*Tg10g06570.t1*	10	5	**+**	181	8.109	20,447.95	MIKC	FUL	6 × 10^−45^	SQUA (A)
*TgMADS42*	*Tg11g05170.t1*	11	5	**+**	185	8.103	21,076.69	MIKC	FUL	2 × 10^−51^	SQUA (A)
*TgMADS43*	*Tg11g11530.t1*	11	8	**−**	259	7.997	29,823.83	MIKC	AP1	1 × 10^−105^	SQUA (A)
*TgMADS44*	*Tg11g11540.t1*	11	8	**−**	238	8.408	27,297.68	MIKC	SEP4	8 × 10^−89^	SEP (E)
*TgMADS45*	*Tg11g14500.t1* *	11	7	**−**	187	8.599	21,300.46	MIKC	MAF3	8 × 10^−38^	FLC
*TgMADS46*	*Tg11g14510.t1*	11	6	**−**	214	8.399	24,197.23	MIKC	SEP3	1 × 10^−111^	SEP (E)
*TgMADS47*	*Tg12g09890.t1* *	12	6	**+**	206	8.455	23,037.09	MIKC	AGL24	5 × 10^−58^	STMADS11 (SVP)
*TgMADS48*	*Tg12g10450.t1* *	12	14	**−**	400	8.94	45,465.40	MIKC	ANR1	5 × 10^−42^	AGL17 (ANR1)
*TgMADS49*	*Tg12g10710.t1* *	12	12	**+**	342	9.122	39,025.15	MIKC	ANR1	1 × 10^−42^	AGL17 (ANR1)
*TgMADS50*	*Tg12g10730.t1*	12	7	**+**	234	8.587	26,692.53	MIKC	AGL21	1 × 10^−87^	AGL17 (ANR1)
*TgMADS51*	*Tg12g10740.t1* *	12	7	**+**	246	7.537	27,560.23	MIKC	AGL16	1 × 10^−72^	AGL17 (ANR1)
*TgMADS52*	*Tg12g11440.t1*	12	1	**−**	332	5.971	36,820.75	M type	AGL62	2 × 10^−38^	α
*TgMADS53*	*Tg12g11450.t1*	12	1	**−**	338	5.775	36,382.70	M type	AGL62	7 × 10^−41^	α
*TgMADS54*	*Tg12g11460.t1*	12	1	**−**	218	8.785	24,618.95	M type	AGL23	4 × 10^−35^	α
*TgMADS55*	*Tg12g11470.t1*	12	1	**−**	236	8.327	25,952.45	M type	AGL40	2 × 10^−35^	α
*TgMADS56*	*Tg12g11480.t1*	12	1	**−**	286	8.281	31,056.86	M type	AGL28	1 × 10^−29^	α
*TgMADS57*	*Tg13g04470.t1*	13	7	**+**	272	9.361	31,573.91	MIKC	SHP1	1 × 10^−111^	AG (C/D)
*TgMADS58*	*Tg13g08100.t1*	13	1	**+**	157	9.188	18,603.25	M type	AGL45	7 × 10^−17^	γ
*TgMADS59*	*Tg13g08220.t1*	13	1	**−**	188	8.956	21,716.79	M type	AGL45	4 × 10^−20^	γ
*TgMADS60*	*Tg14g04500.t1*	14	1	**+**	180	8.876	20,944.87	M type	AGL29	3 × 10^−30^	α
*TgMADS61*	*Tg15g03960.t1* *	15	7	**−**	217	7.666	24,841.28	MIKC	MAF2	6 × 10^−33^	FLC
*TgMADS62*	*Tg15g08040.t1* *	15	6	**+**	214	8.57	24,725.49	MIKC	FYF	4 × 10^−64^	SOC1/TM3
*TgMADS63*	*Tg15g12120.t1*	15	6	**+**	213	7.963	24,092.98	MIKC	SEP4	4 × 10^−60^	SEP (E)
*TgMADS64*	*Tg15g12130.t1*	15	5	**+**	187	6.481	21,347.13	MIKC	AP1	1 × 10^−60^	SQUA (A)
*TgMADS65*	*Tg16g04330.t1*	16	1	**−**	169	5.426	18,449.26	M type	AGL61	1 × 10^−16^	α
*TgMADS66*	*Tg16g06210.t1* *	16	8	**+**	208	8.539	24,148.02	MIKC	AGL19	3 × 10^−55^	SOC1/TM3
*TgMADS67*	*Tg16g08830.t1*	16	8	**+**	202	9.321	23,463.77	MIKC	TM8	9 × 10^−93^	TM8
*TgMADS68*	*Tg16g13620.t1*	16	1	**+**	341	8.465	39,207.77	M type	AGL47	8 × 10^−23^	γ
*TgMADS69*	*Tg17g01590.t1*	17	9	**−**	244	8.824	28,212.51	MIKC	AG	1 × 10^−101^	AG (C/D)
*TgMADS70*	*Tg17g11550.t1*	17	5	**+**	196	8.399	22,728.76	MIKC	AG	4 × 10^−69^	AG (C/D)
*TgMADS71*	*Tg17g14050.t1* *	17	6	**−**	204	5.185	22,642.22	MIKC	AGL104	1 × 10^−29^	MIKC *
*TgMADS72*	*Tg18g00180.t1*	18	6	**+**	274	8.956	31,067.39	MIKC	STK	2 × 10^−87^	AG (C/D)
*TgMADS73*	*Tg18g03780.t1*	18	5	**−**	181	9.068	21,234.95	MIKC	AP3	7 × 10^−55^	DEF/GLO (B)
*TgMADS74*	*Tg18g12570.t1*	18	1	**−**	180	5.239	19,540.09	M type	AGL62	6 × 10^−18^	α
*TgMADS75*	*TgUn065g00050.t1*	Un65	1	**−**	180	5.239	19,584.10	M type	AGL62	2 × 10^−17^	α
*TgMADS76*	*TgUn001g00120.t1*	Un001	7	**+**	214	8.71	24,498.73	MIKC	AGL19	2 × 10^−71^	SOC1/TM3
*TgMADS77*	*TgUn001g00290.t1*	Un001	7	**−**	214	8.71	24,498.73	MIKC	AGL19	2 × 10^−71^	SOC1/TM3
*TgMADS78*	*TgUn357g00010.t1*	Un357	5	**−**	181	8.109	20,447.95	MIKC	FUL	6 × 10^−45^	SQUA (A)
*TgMADS79*	*TgUn447g00010.t1* *^Δ^	Un447	2 *	**−**	75 *	9.688	8,350.45	MIKC	ANR1	2 × 10^−35^	AGL17 (ANR1)
*TgMADS80*	*TgUn530g00010.t1*	Un530	1	**+**	306	8.864	33,279.50	M type	AGL62	5 × 10^−39^	α
*TgMADS81*	*TgUn530g00020.t1*	Un530	1	**+**	236	8.327	25,910.37	M type	AGL62	3 × 10^−42^	α
*TgMADS82*	*TgUn530g00030.t1*	Un530	1	**+**	218	8.785	24,604.93	M type	AGL23	6 × 10^−35^	α
*TgMADS83*	*TgUn530g00050.t1*	Un530	2	**+**	300	7.422	34,065.09	M type	AGL62	3 × 10^−38^	α
*TgMADS84*	*TgUn613g00040.t1* *	Un613	8	**−**	285	8.241	32,590.56	MIKC	AG	1 × 10^−103^	AG (C/D)
*TgMADS85*	*TgUn699g00040.t1*	Un699	1	**+**	332	5.971	36,824.74	M type	AGL62	2 × 10^−38^	α
*TgMADS86*	*TgUn699g00020.t1*	Un699	1	**+**	191	8.978	21,292.71	M type	AGL62	3 × 10^−40^	α
*TgMADS87*	*TgUn766g00020.t1*	Un766	2	**−**	237	8.579	27,249.67	MIKC	FUL	6 × 10^−68^	SQUA (A)

* Gene annotation was corrected. ^Δ^ Gene is partial. Chr = Chromosome. *TM8* gene is present in *S. indicum* but not in *A. thaliana*.

## Data Availability

The original contributions presented in this study are included in the article/Supplementary Material. Further inquiries can be directed to the corresponding author.

## Supplementary Materials

The following supporting information can be downloaded at: https://www.mdpi.com/article/10.3390/genes17020124/s1, A list of TgMADS peptide sequences is available in the Supplementary Data. The following tables are available in the supplementary data: Table S1: Full consensus sequences HMMR3 score; Table S2: Tandemly repeated genes; and Table S3: Segmental MicroSynteny analysis results.

## References

[1] Harley R.M., Kadereit J.W. (2004). Labiatae. Flowering Plants·Dicotyledons: Lamiales (Except Acanthaceae Including Avicenniaceae).

[2] Mabberley D.J. (2017). Mabberley’s Plant-Book: A Portable Dictionary of Plants, Their Classification and Uses.

[3] Pandey D., Brown C. (2000). Teak: A Global Overview. Unasylva.

[4] Nidavani R.B., Mahalakshmi A. (2014). Pharmacology of *Tectona grandis* Linn.: Short review. Int. J. Pharmacogn. Phytochem. Res..

[5] Vyas P., Yadav D.K., Khandelwal P. (2019). *Tectona grandis* (teak)—A review on its phytochemical and therapeutic potential. Nat. Prod. Res..

[6] Asdaq S.M.B., Nayeem N., Alam M.T., Alaqel S.I., Imran M., Hassan E.-W.E., Rabbani S.I. (2022). *Tectona grandis* Lf: A comprehensive review on its patents, chemical constituents, and biological activities. Saudi J. Biol. Sci..

[7] Khera N., Bhargava S. (2013). Phytochemical and pharmacological evaluation of *Tectona grandis* Linn. Int. J. Pharm. Pharm. Sci..

[8] Kolli P.K., Obbalareddy S., Yejella R.P., Athili L.D., Ponnada S. (2022). A Review on *Tectona grandis*. Int. J. Res. Pharm. Chem..

[9] Singh N., Dixit K., Kumar K. (2024). Pharmacological, and Phytochemical Profile of *Tectona grandis* linn (Verbenaceae)—A Comprehensive Review. Afr. J. Bio. Sc..

[10] Palanisamy K., Hegde M., Yi J.-S. (2009). Teak (*Tectona grandis* Linn. f.): A renowned commercial timber species. J. For. Environ. Sci..

[11] Sahu S.K., Liu M., Chen Y., Gui J., Fang D., Chen X., Yang T., He C., Cheng L., Yang J. (2023). Chromosome-scale genomes of commercial timber trees (*Ochroma pyramidale*, *Mesua ferrea*, and *Tectona grandis*). Sci. Data.

[12] Zhao D., Hamilton J.P., Bhat W.W., Johnson S.R., Godden G.T., Kinser T.J., Boachon B., Dudareva N., Soltis D.E., Soltis P.S. (2019). A chromosomal-scale genome assembly of *Tectona grandis* reveals the importance of tandem gene duplication and enables discovery of genes in natural product biosynthetic pathways. Gigascience.

[13] Yasodha R., Vasudeva R., Balakrishnan S., Sakthi A.R., Abel N., Binai N., Rajashekar B., Bachpai V.K.W., Pillai C., Dev S.A. (2018). Draft genome of a high value tropical timber tree, Teak (*Tectona grandis* L. f): Insights into SSR diversity, phylogeny and conservation. DNA Res..

[14] Matias Hurtado F.M., Pinto M.d.S., Oliveira P.N.d., Riaño-Pachón D.M., Inocente L.B., Carrer H. (2019). Analysis of NAC domain transcription factor genes of *Tectona grandis* Lf involved in secondary cell wall deposition. Genes.

[15] Maisuria H.J., Dhaduk H.L., Kumar S., Sakure A.A., Thounaojam A.S. (2022). Teak population structure and genetic diversity in Gujarat, India. Curr. Plant Biol..

[16] Nurtjahjaningsih I.L.G., Rimbawanto A., Fauzi M.A., Dormontt E.E., Lowe A.J., Hendrati R.L., Baskorowati L., Susanto M., Sulistiadi H.B.S., Mashudi (2023). Assessing the genetic structure of teak from Southeast Sulawesi and its implication for genetic conservation and utilization in Indonesia. For. Sci. Technol..

[17] Dania V.O., Osunlaja O.A., Igwe D.O. (2020). Evaluation of genetic diversity using inter-simple sequence repeat markers and effect on the severity of leaf blight disease of teak (*Tectona grandis* L.). J. Sustain. For..

[18] Dos Anjos I.V., Gilio T.A.S., Amorim A.F.S., de Jesus J.G., Chimello A.M., Takizawa F.H., Araujo K.L., Neves L.G. (2023). Reassessing the genetic variability of *Tectona grandis* through high-throughput genotyping: Insights on its narrow genetic base. PLoS ONE.

[19] Galeano E., Vasconcelos T.S., Novais de Oliveira P., Carrer H. (2019). Physiological and molecular responses to drought stress in teak (*Tectona grandis* Lf). PLoS ONE.

[20] Huang G., Liang K., Zhou Z., Yang G., Muralidharan E.M. (2019). Variation in photosynthetic traits and correlation with growth in teak (*Tectona grandis* Linn.) Clones. Forests.

[21] Pachas A., Sakanphet S., Midgley S., Dieters M. (2019). Teak (*Tectona grandis*) silviculture and research: Applications for smallholders in Lao PDR. Aust. For..

[22] Shore P., Sharrocks A.D. (1995). The MADS-box family of transcription factors. Eur. J. Biochem..

[23] Li C., Wang Y., Xu L., Nie S., Chen Y., Liang D., Sun X., Karanja B.K., Luo X., Liu L. (2016). Genome-wide characterization of the MADS-box gene family in radish (*Raphanus sativus* L.) and assessment of its roles in flowering and floral organogenesis. Front. Plant Sci..

[24] Purugganan M.D., Rounsley S.D., Schmidt R.J., Yanofsky M.F. (1995). Molecular evolution of flower development: Diversification of the plant MADS-box regulatory gene family. Genetics.

[25] Dias B.F.d.O., Simões-Araújo J.L., Russo C.A., Margis R., Alves-Ferreira M. (2005). Unravelling MADS-box gene family in *Eucalyptus* spp.: A starting point to an understanding of their developmental role in trees. Genet. Mol. Biol..

[26] Ma J., Yang Y., Luo W., Yang C., Ding P., Liu Y., Qiao L., Chang Z., Geng H., Wang P. (2017). Genome-wide identification and analysis of the MADS-box gene family in bread wheat (*Triticum aestivum* L.). PLoS ONE.

[27] Zhao D., Chen Z., Xu L., Zhang L., Zou Q. (2021). Genome-wide analysis of the MADS-box gene family in maize: Gene structure, evolution, and relationships. Genes.

[28] Raza Q., Riaz A., Atif R.M., Hussain B., Rana I.A., Ali Z., Budak H., Alaraidh I.A. (2022). Genome-wide diversity of MADS-box genes in bread wheat is associated with its rapid global adaptability. Front. Genet..

[29] Wei M., Wang Y., Pan R., Li W. (2018). Genome-Wide Identification and Characterization of MADS-box Family Genes Related to Floral Organ Development and Stress Resistance in *Hevea brasiliensis* Müll. Arg. Forests.

[30] Hao X., Fu Y., Zhao W., Liu L., Bade R., Hasi A., Hao J. (2016). Genome-wide identification and analysis of the MADS-box gene family in melon. J. Am. Soc. Hortic. Sci..

[31] de Campos Rume G., de Oliveira R.R., Ribeiro T.H.C., Chalfun-Júnior A. (2023). Genome-wide and expression analyses of MADS-box genes in the tetraploid *Coffea arabica* L. and its diploid parental subgenomes. Plant Gene.

[32] Gao H., Wang Z., Li S., Hou M., Zhou Y., Zhao Y., Li G., Zhao H., Ma H. (2018). Genome-wide survey of potato MADS-box genes reveals that StMADS1 and StMADS13 are putative downstream targets of tuberigen StSP6A. BMC Genom..

[33] Grimplet J., Martinez-Zapater J.M., Carmona M.J. (2016). Structural and functional annotation of the MADS-box transcription factor family in grapevine. BMC Genom..

[34] Lu J., Wu H., Pitt D.M., Liu X., Song X., Yuan H., Ma Y., Li S., Zang Z., Zhang J. (2024). Identification and characterization of MADS-box gene family in flax, *Linum usitatissimum* L. and its role under abiotic stress. iScience.

[35] Kapazoglou A., Engineer C., Drosou V., Kalloniati C., Tani E., Tsaballa A., Kouri E.D., Ganopoulos I., Flemetakis E., Tsaftaris A.S. (2012). The study of two barley type I-like MADS-box genes as potential targets of epigenetic regulation during seed development. BMC Plant Biol..

[36] Sheng X.-G., Zhao Z.-Q., Wang J.-S., Yu H.-F., Shen Y.-S., Zeng X.-Y., Gu H.-H. (2019). Genome wide analysis of MADS-box gene family in *Brassica oleracea* reveals conservation and variation in flower development. BMC Plant Biol..

[37] Gramzow L., Theissen G. (2010). A hitchhiker’s guide to the MADS world of plants. Genome Biol..

[38] Bemer M., Gordon J., Weterings K., Angenent G.C. (2010). Divergence of Recently Duplicated M γ-Type MADS-Box Genes in *Petunia*. Mol. Biol. Evol..

[39] Masiero S., Colombo L., Grini P.E., Schnittger A., Kater M.M. (2011). The emerging importance of type I MADS box transcription factors for plant reproduction. Plant Cell.

[40] Gramzow L., Weilandt L., Theissen G. (2014). MADS goes genomic in conifers: Towards determining the ancestral set of MADS-box genes in seed plants. Ann. Bot..

[41] Ambrose B.A., Smalls T.L., Zumajo-Cardona C. (2021). All type II classic MADS-box genes in the lycophyte *Selaginella moellendorffii* are broadly yet discretely expressed in vegetative and reproductive tissues. Evol. Dev..

[42] Thangavel G., Nayar S. (2018). A survey of MIKC type MADS-box genes in non-seed plants: Algae, bryophytes, lycophytes and ferns. Front. Plant Sci..

[43] Nam J., Kim J., Lee S., An G., Ma H., Nei M. (2004). Type I MADS-box genes have experienced faster birth-and-death evolution than type II MADS-box genes in angiosperms. Proc. Natl. Acad. Sci. USA.

[44] Theißen G. (2001). Development of floral organ identity: Stories from the MADS house. Curr. Opin. Plant Biol..

[45] Ditta G., Pinyopich A., Robles P., Pelaz S., Yanofsky M.F. (2004). The SEP4 Gene of *Arabidopsis thaliana* Functions in Floral Organ and Meristem Identity. Curr. Biol..

[46] Mendes M.A., Guerra R.F., Berns M.C., Manzo C., Masiero S., Finzi L., Kater M.M., Colombo L. (2013). MADS Domain Transcription Factors Mediate Short-Range DNA Looping That Is Essential for Target Gene Expression in *Arabidopsis*. Plant Cell.

[47] Gramzow L., Tessari C., Rümpler F., Theißen G. (2023). Deep evolution of MADS-box genes in Archaeplastida. bioRxiv.

[48] Sneddon T.P., Li P., Edmunds S.C. (2012). GigaDB: Announcing the GigaScience database. GigaScience.

[49] Mistry J., Chuguransky S., Williams L., Qureshi M., Salazar G.A., Sonnhammer E.L.L., Tosatto S.C., Paladin L., Raj S., Richardson L.J. (2020). Pfam: The protein families database in 2021. Nucleic Acids Res..

[50] Letunic I., Khedkar S., Bork P. (2020). SMART: Recent updates, new developments and status in 2020. Nucleic Acids Res..

[51] Sigrist C.J.A., Cerutti L., Hulo N., Gattiker A., Falquet L., Pagni M., Bairoch A., Bucher P. (2002). PROSITE: A documented database using patterns and profiles as motif descriptors. Brief. Bioinform..

[52] Solovyev V., Kosarev P., Seledsov I., Vorobyev D. (2006). Automatic annotation of eukaryotic genes, pseudogenes and promoters. Genome Biol..

[53] Tian F., Yang D.-C., Meng Y.-Q., Jin J., Gao G. (2019). PlantRegMap: Charting functional regulatory maps in plants. Nucleic Acids Res..

[54] Gasteiger E., Hoogland C., Gattiker A., Duvaud S.e., Wilkins M.R., Appel R.D., Bairoch A., Walker J.M. (2005). Protein Identification and Analysis Tools on the ExPASy Server. The Proteomics Protocols Handbook.

[55] Chen C., Chen H., Zhang Y., Thomas H.R., Frank M.H., He Y., Xia R. (2020). TBtools: An Integrative Toolkit Developed for Interactive Analyses of Big Biological Data. Mol. Plant.

[56] Qiao X., Li Q., Yin H., Qi K., Li L., Wang R., Zhang S., Paterson A.H. (2019). Gene duplication and evolution in recurring polyploidization–diploidization cycles in plants. Genome Biol..

[57] Hu B., Jin J., Guo A.Y., Zhang H., Luo J., Gao G. (2014). GSDS 2.0: An upgraded gene feature visualization server. Bioinformatics.

[58] Bailey T.L., Boden M., Buske F.A., Frith M., Grant C.E., Clementi L., Ren J., Li W.W., Noble W.S. (2009). MEME Suite: Tools for motif discovery and searching. Nucleic Acids Res..

[59] Jin J., Tian F., Yang D.C., Meng Y.Q., Kong L., Luo J., Gao G. (2016). PlantTFDB 4.0: Toward a central hub for transcription factors and regulatory interactions in plants. Nucleic Acids Res..

[60] Okonechnikov K., Golosova O., Fursov M., UGENE Team (2012). Unipro UGENE: A unified bioinformatics toolkit. Bioinformatics.

[61] Kumar S., Stecher G., Suleski M., Sanderford M., Sharma S., Tamura K. (2024). MEGA12: Molecular Evolutionary Genetic Analysis Version 12 for Adaptive and Green Computing. Mol. Biol. Evol..

[62] Abramson J., Adler J., Dunger J., Evans R., Green T., Pritzel A., Ronneberger O., Willmore L., Ballard A.J., Bambrick J. (2024). Accurate structure prediction of biomolecular interactions with AlphaFold 3. Nature.

[63] Abraham M., Alekseenko A., Andrews B., Bauer P., Bergh C., Bird H., Briand E., Brown A., Chen Y., Doijade M. (2026). GROMACS 2026.0 Source Code, version 2026.0.

[64] Wei X., Wang L., Yu J., Zhang Y., Li D., Zhang X. (2015). Genome-wide identification and analysis of the MADS-box gene family in sesame. Gene.

[65] Alhindi T., Al-Abdallat A.M. (2021). Genome-Wide Identification and Analysis of the MADS-Box Gene Family in American Beautyberry (*Callicarpa americana*). Plants.

[66] Alhindi T., Zhang Z., Ruelens P., Coenen H., Degroote H., Iraci N., Geuten K. (2017). Protein interaction evolution from promiscuity to specificity with reduced flexibility in an increasingly complex network. Sci. Rep..

[67] Puranik S., Acajjaoui S., Conn S., Costa L., Conn V., Vial A., Marcellin R., Melzer R., Brown E., Hart D. (2014). Structural Basis for the Oligomerization of the MADS Domain Transcription Factor SEPALLATA3 in *Arabidopsis*. Plant Cell.

[68] Qiu Y., Li Z., Walther D., Kohler C. (2023). Updated Phylogeny and Protein Structure Predictions Revise the Hypothesis on the Origin of MADS-box Transcription Factors in Land Plants. Mol. Biol. Evol..

[69] Yoshida T., Kawabe A. (2013). Importance of gene duplication in the evolution of genomic imprinting revealed by molecular evolutionary analysis of the type I MADS-box gene family in *Arabidopsis* species. PLoS ONE.

[70] Upadhyay A.K., Chacko A.R., Gandhimathi A., Ghosh P., Harini K., Joseph A.P., Joshi A.G., Karpe S.D., Kaushik S., Kuravadi N. (2015). Genome sequencing of herb Tulsi (*Ocimum tenuiflorum*) unravels key genes behind its strong medicinal properties. BMC Plant Biol..

[71] Shu Y., Yu D., Wang D., Guo D., Guo C. (2013). Genome-wide survey and expression analysis of the MADS-box gene family in soybean. Mol. Biol. Rep..

[72] Kagale S., Koh C., Nixon J., Bollina V., Clarke W.E., Tuteja R., Spillane C., Robinson S.J., Links M.G., Clarke C. (2014). The emerging biofuel crop *Camelina sativa* retains a highly undifferentiated hexaploid genome structure. Nat. Commun..

[73] Parenicova L., de Folter S., Kieffer M., Horner D.S., Favalli C., Busscher J., Cook H.E., Ingram R.M., Kater M.M., Davies B. (2003). Molecular and Phylogenetic Analyses of the Complete MADS-Box Transcription Factor Family in *Arabidopsis* : New Openings to the MADS World. Plant Cell.

[74] Theißen G., Rümpler F., Gramzow L. (2018). Array of MADS-Box Genes: Facilitator for Rapid Adaptation?. Trends Plant Sci..

[75] Cannon S.B., Mitra A., Baumgarten A., Young N.D., May G. (2004). The roles of segmental and tandem gene duplication in the evolution of large gene families in *Arabidopsis thaliana*. BMC Plant Biol.

[76] Kofuji R., Sumikawa N., Yamasaki M., Kondo K., Ueda K., Ito M., Hasebe M. (2003). Evolution and Divergence of the MADS-Box Gene Family Based on Genome-Wide Expression Analyses. Mol. Biol. Evol..

[77] Lemons D., McGinnis W. (2006). Genomic Evolution of *HOX* Gene Clusters. Science.

[78] Ng M., Yanofsky M.F. (2001). Function and evolution of the plant MADS-box gene family. Nat. Rev. Genet..

[79] Geeta R. (2003). The origin and maintenance of nuclear endosperms: Viewing development through a phylogenetic lens. Proc. R. Soc. B Biol. Sci..

[80] Płachno B.J., Świątek P., Sas-Nowosielska H., Kozieradzka-Kiszkurno M. (2013). Organisation of the endosperm and endosperm–placenta syncytia in bladderworts (*Utricularia*, Lentibulariaceae) with emphasis on the microtubule arrangement. Protoplasma.

[81] Coenen H., Viaene T., Vandenbussche M., Geuten K. (2018). TM8 represses developmental timing in *Nicotiana benthamiana* and has functionally diversified in angiosperms. BMC Plant Biol..

[82] Daminato M., Masiero S., Resentini F., Lovisetto A., Casadoro G. (2014). Characterization of TM8, a MADS-box gene expressed in tomato flowers. BMC Plant Biol..

[83] Yang Y., Fanning L., Jack T. (2003). The K domain mediates heterodimerization of the *Arabidopsis* floral organ identity proteins, APETALA3 and PISTILLATA. Plant J..

[84] Airoldi C.A., McKay M., Davies B. (2015). MAF2 Is Regulated by Temperature-Dependent Splicing and Represses Flowering at Low Temperatures in Parallel with FLM. PLoS ONE.

[85] Ratcliffe O.J., Kumimoto R.W., Wong B.J., Riechmann J.L. (2003). Analysis of the *Arabidopsis* MADS AFFECTING FLOWERING Gene Family: MAF2 Prevents Vernalization by Short Periods of Cold. Plant Cell.

[86] Madrigal Y., Alzate J.F., Pabon-Mora N. (2024). Evolution of major flowering pathway integrators in Orchidaceae. Plant Reprod..

[87] Immink R.G., Tonaco I.A., de Folter S., Shchennikova A., van Dijk A.D., Busscher-Lange J., Borst J.W., Angenent G.C. (2009). SEPALLATA3: The ‘glue’ for MADS box transcription factor complex formation. Genome Biol..

[88] Kwantes M., Liebsch D., Verelst W. (2011). How MIKC* MADS-Box Genes Originated and Evidence for Their Conserved Function Throughout the Evolution of Vascular Plant Gametophytes. Mol. Biol. Evol..

[89] Castelán-Muñoz N., Herrera J., Cajero-Sánchez W., Arrizubieta M., Trejo C., García-Ponce B., Sánchez M.D.L.P., Álvarez-Buylla E.R., Garay-Arroyo A. (2019). MADS-Box Genes Are Key Components of Genetic Regulatory Networks Involved in Abiotic Stress and Plastic Developmental Responses in Plants. Front. Plant Sci..

[90] Yin W., Hu Z., Hu J., Zhu Z., Yu X., Cui B., Chen G. (2017). Tomato (*Solanum lycopersicum*) MADS-box transcription factor SlMBP8 regulates drought, salt tolerance and stress-related genes. Plant Growth Regul..

[91] Godden G.T., Kinser T.J., Soltis P.S., Soltis D.E. (2019). Phylotranscriptomic Analyses Reveal Asymmetrical Gene Duplication Dynamics and Signatures of Ancient Polyploidy in Mints. Genome Biol. Evol..

