# Supplementary material for: Genome-Wide Identification and Analysis of the MADS-Box Gene Family in Tectona grandis (Teak), a Member of the Lamiaceae Family
Source: Genes (Basel). 2026 Jan 25;17(2):124. doi: 10.3390/genes17020124 (PMC12940651; doi:10.3390/genes17020124)
Supplement: Supplementary file 1 [file genes-17-00124-s001.zip › Supplementary Data.pdf]

Genome-wide Identification and Analysis of the MADS-box Gene Family in Tectona grandis (Teak), a Member of the Lamiaceae Family

(Supplementary Data)

Table S1: Full consensus sequences HMMR3 score.

Gene Name	Gene ID	E-value	Score	Bias
TgMADS01	Tg01g02120.t1	3.6e-25	88.7	0.2
TgMADS02	Tg01g02320.t1	3.6e-25	88.7	0.2
TgMADS03	Tg02g16980.t1	2e-26	92.7	0.2
TgMADS04	Tg02g17630.t1*	9.3e-21	74.6	0.3
TgMADS05	Tg02g17650.t1*	4.4e-22	78.8	0.1
TgMADS06	Tg03g08370.t1	1.5e-23	83.5	0.2
TgMADS07	Tg03g12830.t1	2e-15	57.5	0.3
TgMADS08	Tg03g12970.t1	1.9e-26	92.8	0.2
TgMADS09	Tg03g13060.t1	4.9e-22	78.7	0.0
TgMADS10	Tg03g18430.t1	7.7e-26	90.9	0.1
TgMADS11	Tg03g18920.t1	1.1e-25	90.3	0.1
TgMADS12	Tg04g01300.t1	9e-15	55.4	0.0
TgMADS13	Tg05g03610.t1	4.7e-24	85.1	0.4
TgMADS14	Tg05g04300.t1	1.4e-20	74.0	0.0
TgMADS15	Tg05g05250.t1	1.4e-20	74.0	0.0
TgMADS16	Tg05g13890.t1	9.8e-26	90.5	0.6
TgMADS17	Tg05g16950.t1	3.6e-27	95.1	0.4
TgMADS18	Tg05g17300.t1	3.6e-27	95.1	0.4
TgMADS19	Tg06g03090.t1	2.1e-27	95.9	0.4
TgMADS20	Tg06g03840.t1	1.2e-13	51.8	0.0
TgMADS21	Tg06g05880.t1	1.5e-16	61.2	0.1
TgMADS22	Tg06g08110.t1*	5.8e-23	81.6	0.2
TgMADS23	Tg06g09940.t1	9.6e-26	90.5	0.1
TgMADS24	Tg06g11190.t1	3.9e-18	66.2	0.2
TgMADS25	Tg12gnew.t1*	NA	NA	**NA**
TgMADS26	Tg07g06420.t1	2.2e-24	86.2	0.5
TgMADS27	Tg07g08930.t1*	4.8e-26	91.5	0.1
TgMADS28	Tg07g13640.t1	3.8e-26	91.8	0.2
TgMADS29	Tg07g14620.t1*	1.2e-19	71.1	0.7
TgMADS30	Tg08g10570.t1	5.8e-26	91.3	0.1
TgMADS31	Tg08g12890.t1	2.8e-13	50.6	0.2
TgMADS32	Tg08g12940.t1	3.6e-15	56.7	0.0

TgMADS33	Tg08g14560.t1	2.1e-20	73.4	0.4
TgMADS34	Tg08g14570.t1	6.6e-25	87.9	0.3
TgMADS35	Tg09g00890.t1	3.8e-26	91.8	0.3
TgMADS36	Tg09g00900.t1*	1.7e-25	89.8	0.3
TgMADS37	Tg09g02970.t1	4.4e-14	53.2	0.1
TgMADS38	Tg09g10160.t1	3e-25	89.0	0.2
TgMADS39	Tg09g12140.t1*	5.9e-18	65.6	0.3
TgMADS40	Tg10g05610.t1	5.7e-17	62.5	0.0
TgMADS41	Tg10g06570.t1	4.1e-23	82.1	0.1
TgMADS42	Tg11g05170.t1	2.5e-25	89.2	0.2
TgMADS43	Tg11g11530.t1	1.2e-25	90.2	0.2
TgMADS44	Tg11g11540.t1	1.5e-26	93.1	0.4
TgMADS45	Tg11g14500.t1*	6.4e-25	87.9	0.1
TgMADS46	Tg11g14510.t1	3.6e-26	91.9	0.3
TgMADS47	Tg12g09890.t1*	4.4e-25	88.4	0.2
TgMADS48	Tg12g10450.t1*	1.2e-26	93.5	0.1
TgMADS49	Tg12g10710.t1*	1.2e-26	93.5	0.1
TgMADS50	Tg12g10730.t1	7.9e-23	81.2	0.2
TgMADS51	Tg12g10740.t1*	2.7e-24	85.9	0.1
TgMADS52	Tg12g11440.t1	7.2e-24	84.5	0.1
TgMADS53	Tg12g11450.t1	3.4e-23	82.4	0.0
TgMADS54	Tg12g11460.t1	1.1e-24	87.2	0.3
TgMADS55	Tg12g11470.t1	3.9e-22	79.0	0.4
TgMADS56	Tg12g11480.t1	8.7e-24	84.3	0.1
TgMADS57	Tg13g04470.t1	2.9e-26	92.2	0.2
TgMADS58	Tg13g08100.t1	7.3e-16	58.9	0.1
TgMADS59	Tg13g08220.t1	1.5e-15	57.9	0.0
TgMADS60	Tg14g04500.t1	1.7e-19	70.5	0.2
TgMADS61	Tg15g03960.t1*	2.9e-24	85.8	0.1
TgMADS62	Tg15g08040.t1*	6.2e-28	97.5	0.0
TgMADS63	Tg15g12120.t1	2e-26	92.7	0.3
TgMADS64	Tg15g12130.t1	5.2e-26	91.4	0.3
TgMADS65	Tg16g04330.t1	8.3e-15	55.5	0.1
TgMADS66	Tg16g06210.t1*	1.7e-27	96.2	0.2
TgMADS67	Tg16g08830.t1	3.3e-27	95.2	0.0
TgMADS68	Tg16g13620.t1	3.8e-14	53.4	0.1
TgMADS69	Tg17g01590.t1	2.7e-26	92.3	0.2
TgMADS70	Tg17g11550.t1	5.6e-26	91.3	0.3
TgMADS71	Tg17g14050.t1*	1.2e-18	67.8	0.0
TgMADS72	Tg18g00180.t1	5.5e-26	91.3	0.2
TgMADS73	Tg18g03780.t1	2.7e-17	63.5	0.4

TgMADS74	Tg18g12570.t1	9.2e-15	55.4	0.1
TgMADS75	TgUn065g00050.t1	7.4e-15	55.7	0.3
TgMADS76	TgUn001g00120.t1	1.4e-27	96.4	0.1
TgMADS77	TgUn001g00290.t1	1.4e-27	96.4	0.1
TgMADS78	TgUn357g00010.t1	4.1e-23	82.1	0.1
TgMADS79	TgUn447g00010.t1*	1.2e-26	93.5	0.1
TgMADS80	TgUn530g00010.t1	9.6e-24	84.1	0.1
TgMADS81	TgUn530g00020.t1	4e-22	79.0	0.4
TgMADS82	TgUn530g00030.t1	1.1e-24	87.2	0.3
TgMADS83	TgUn530g00050.t1	6.1e-24	84.8	0.1
TgMADS84	TgUn613g00040.t1*	6.7e-27	94.3	0.2
TgMADS85	TgUn699g00040.t1	7.2e-24	84.5	0.1
TgMADS86	TgUn699g00020.t1	1.3e-23	83.7	0.0
TgMADS87	TgUn766g00020.t1	5.8e-26	91.2	0.2

* Gene annotation has been corrected. *TgMADS25* gene was detected by Fgenesh software.

Table S2: Tandemly Repeated Genes.

Gene 1	Gene 2	Distance bp	Chromosomes
TgMADS33	*TgMADS34*	2660	8
TgMADS35	*TgMADS36*	20745	9
TgMADS43	*TgMADS44*	13109	11
TgMADS45	*TgMADS46*	5373	11
TgMADS50	*TgMADS51*	21906	12
TgMADS52	*TgMADS53*	3355	12
TgMADS53	*TgMADS54*	5843	12
TgMADS54	*TgMADS55*	3563	12
TgMADS55	*TgMADS56*	3423	12
TgMADS63	*TgMADS64*	13363	15
TgMADS80	*TgMADS81*	3611	Un530
TgMADS81	*TgMADS82*	3614	Un530
TgMADS82	*TgMADS83*	8973	Un530
TgMADS86	*TgMADS85*	3368	Un699

Table S3: Segmental MicroSynteny Analysis Results.

Gene 1	Gene 2	Anchor support	Chromosomes
TgMADS08	*TgMADS72*	6	3, 18
TgMADS28	*TgMADS38*	11	7, 9
TgMADS43	*TgMADS64*	6	11, 15
TgMADS44	*TgMADS63*	6	11, 15
TgMADS45	*TgMADS61*	7	11, 15
TgMADS74	*TgMADS75*	8	18, Un65
TgMADS76	*TgMADS77*	12	Un001, Un001
TgMADS10	*TgMADS11*	17	3, 3
TgMADS17	*TgMADS18*	6	5, 5
TgMADS58	*TgMADS59*	6	13, 13
